# Supplementary material for: Voltage Mining for (De)lithiation-Stabilized Cathodes and a Machine Learning Model for Li-Ion Cathode Voltage
Source: ACS Appl Mater Interfaces. 2024 Dec 9;16(50):69379–87. doi: 10.1021/acsami.4c15742 (PMC11660040; doi:10.1021/acsami.4c15742)
Supplement: Supplementary file 1 — am4c15742_si_001.pdf [file am4c15742_si_001.pdf]

Supporting Information

Voltage Mining for (De)lithiation-stabilized Cathodes and a Machine Learning Model for Li-ion Cathode Voltage

Haoming Howard Li,[†] Qian Chen,[‡] Gerbrand Ceder,[†,‡] and Kristin A. Persson*,[†,‡]

†*Department of Material Science and Engineering, University of California, Berkeley, CA 94720, U.S.A.*

‡*Materials Science Division, Lawrence Berkeley National Laboratory, Berkeley, 94720, United States*

E-mail: kapersson@lbl.gov

1 Voltage comparison between DLS and LS materials

In general, DLS cathodes are expected to have lower voltages than LS cathodes. For the lithitaion process described by

$$Li_{x_1}MX + (x_2 - x_1)Li \rightarrow Li_{x_2}MX \tag{S1}$$

the average voltage at low temperatures can be approximated as[1]

$$\bar{V}(x_1, x_2) = -\frac{E(Li_{x_2}MX) - E(Li_{x_1}MX) - (x_2 - x_1)E(Li)}{(x_2 - x_1)F} \tag{S2}$$

where the internal energies of the less lithiated phase $Li_{x_1}MX$, the more lithiated phase $Li_{x_2}MX$ and elemental Li, can be obtained from *ab initio* calculations. We can rewrite this expression in terms of the internal energy of the most stable phase (on the convex hull, denoted as E_{hull}) and E-above-hull (denoted E_{above_hull}) for both $Li_{x_1}MX$ and $Li_{x_2}MX$, as such:

$$\begin{aligned}\bar{V}(x_1, x_2) = &- \frac{(E_{hull}(Li_{x_2}MX) + E_{above\_hull}(Li_{x_2}MX)) - (E_{hull}(Li_{x_1}MX) + E_{above\_hull}(Li_{x_1}MX))}{(x_2 - x_1)F} \\ &+ \frac{(x_2 - x_1)E(Li)}{(x_2 - x_1)F}\end{aligned} \tag{S3}$$

For a pair of DLS and LS systems that share the same chemical evolution $Li_{x_1}R \rightarrow Li_{x_2}R$ during lithiation, we can compare their voltages:

$$\begin{aligned}&\bar{V}^{DLS}(x_1, x_2) - \bar{V}^{LS}(x_1, x_2) = \\ &- \frac{(E_{hull}(Li_{x_2}MX) + E^{DLS}_{above\_hull}(Li_{x_2}MX)) - (E_{hull}(Li_{x_1}MX) + E^{DLS}_{above\_hull}(Li_{x_1}MX))}{(x_2 - x_1)F} \\ &+ \frac{(E_{hull}(Li_{x_2}MX) + E^{LS}_{above\_hull}(Li_{x_2}MX)) - (E_{hull}(Li_{x_1}MX) + E^{LS}_{above\_hull}(Li_{x_1}MX))}{(x_2 - x_1)F}\end{aligned} \tag{S4}$$

where the energetic contributions from elemental Li cancel out. Since the energies of the most stable phase (E_{hull}) for both systems at both compositions are the same, this expression can be further reduced to:

$$\bar{V}^{DLS}(x_1, x_2) - \bar{V}^{LS}(x_1, x_2) \propto$$
$$E^{LS}_{above\_hull}(Li_{x_2}MX) - E^{LS}_{above\_hull}(Li_{x_1}MX) + E^{DLS}_{above\_hull}(Li_{x_1}MX)) - E^{DLS}_{above\_hull}(Li_{x_2}MX) \quad \text{(S5)}$$

By definition, the more lithiated phase for an LS system has a lower E-above-hull that the less lithiated phase, giving rise to $E^{LS}_{above\_hull}(Li_{x_2}MX) - E^{LS}_{above\_hull}(Li_{x_1}MX) < 0$. With a similar argument, we can also obtain that $E^{DLS}_{above\_hull}(Li_{x_1}MX)) - E^{DLS}_{above\_hull}(Li_{x_2}MX) < 0$. This leads to the following conclusion:

$$\bar{V}^{DLS}(x_1, x_2) - \bar{V}^{LS}(x_1, x_2) < 0 \quad \text{(S6)}$$

Therefore in general, DLS cathodes are expected to have lower voltages than LS cathodes.

2 Ranking of redox pairs in one-redox-element entries

The ranking below is obtained from analyzing the average voltages of entries that only contain one redox element. This ranking is used to determine which redox pair is responsible for the voltage in structures with multiple redox elements, in case the bond valence analysis fails.

Cr^{4+}/Cr^{5+}

Cu^{2+}/Cu^{3+}

Co^{2+}/Co^{3+}

V^{4+}/V^{5+}

Fe^{3+}/Fe^{4+}

Ni^{2+}/Ni^{3+}

Mn^{3+}/Mn^{4+}

Co^{3+}/Co^{4+}

Ni^{3+}/Ni^{4+}

Cr^{3+}/Cr^{4+}

Mn^{2+}/Mn^{3+}

Mo^{4+}/Mo^{5+}

Mo^{5+}/Mo^{6+}

V^{3+}/V^{4+}

Mo^{3+}/Mo^{4+}

W^{3+}/W^{4+}

W^{4+}/W^{5+}

Fe^{2+}/Fe^{3+}

Cu^{1+}/Cu^{2+}

W^{5+}/W^{6+}

Cr^{2+}/Cr^{3+}

V^{2+}/V^{3+}

Nb^{4+}/Nb^{5+}

Nb^{3+}/Nb^{4+}

Ti^{3+}/Ti^{4+}

Ti^{2+}/Ti^{3+}

Note that some redox pairs listed here are not present in the voltage distribution plot, because they have fewer than 10 data points in total.

3 Voltage distributions of redox pairs, separated by oxides vs. polyanion groups

The figure below shows the voltage distributions of redox pairs for oxides only.

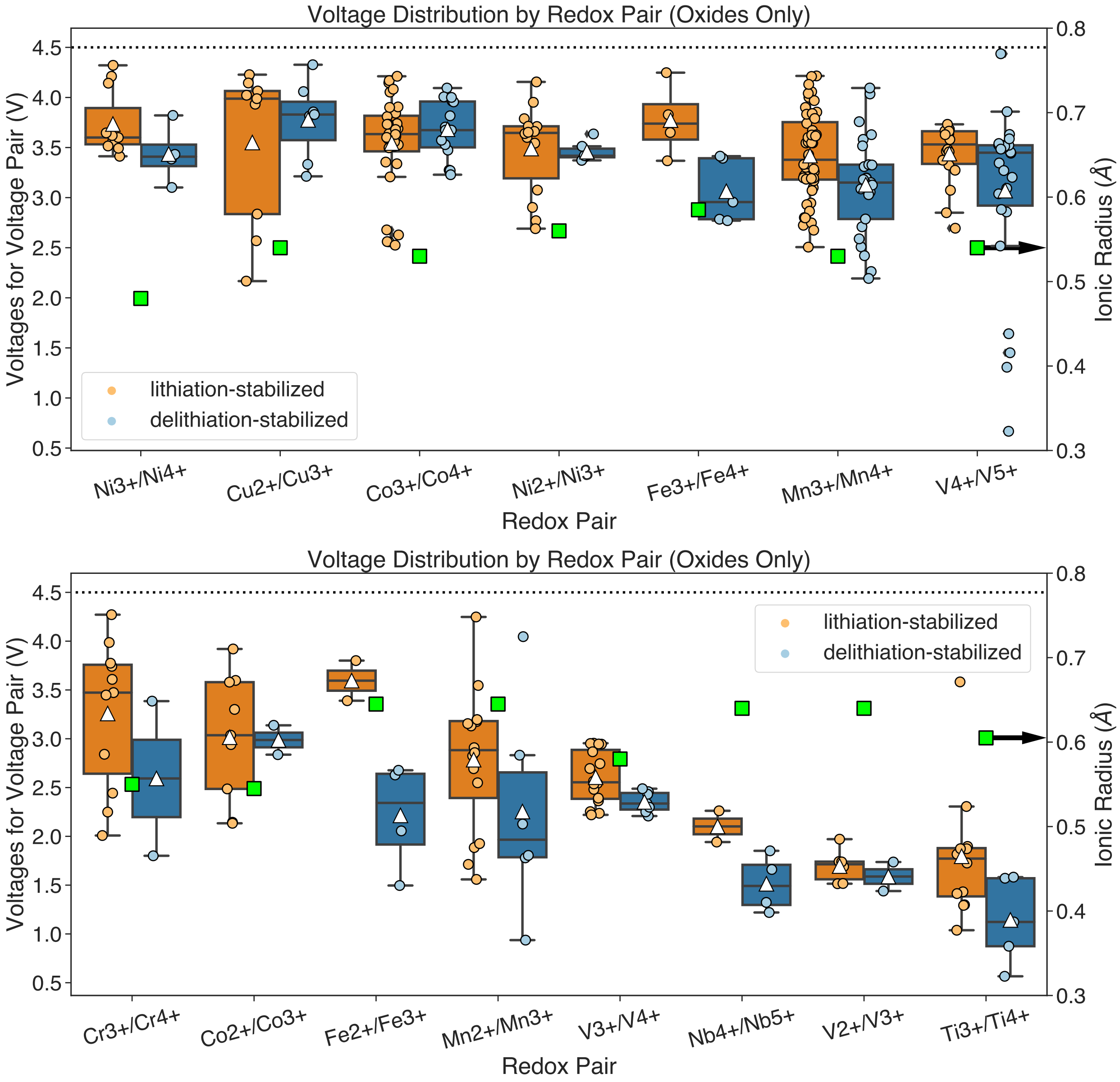

Figure S1: Voltage distribution of redox pairs for oxides only.

The figure below shows the voltage distributions of redox pairs for polyanion groups only.

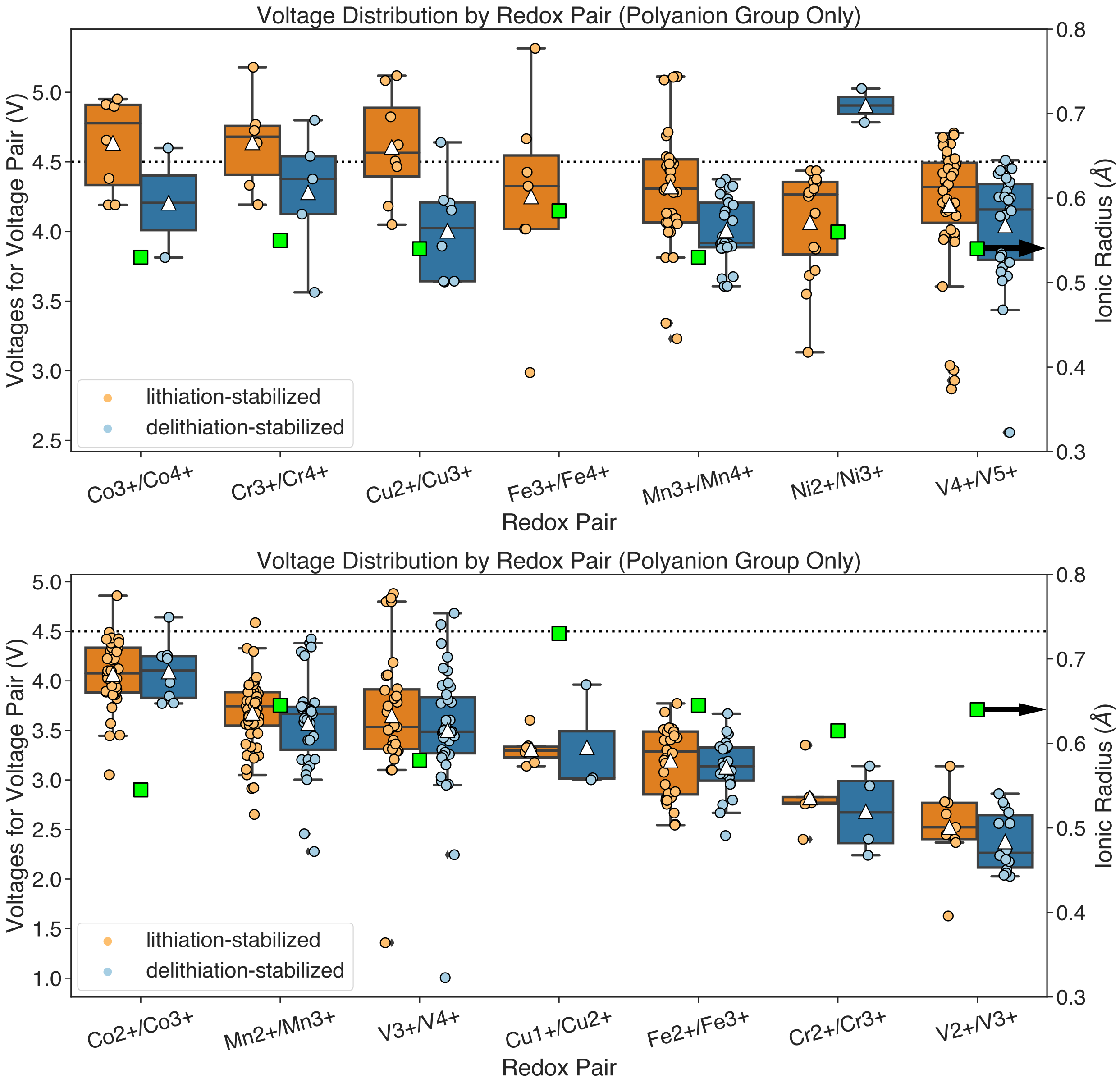

Figure S2: Voltage distribution of redox pairs for polyanion groups only.

References

(1) Aydinol, M. K.; Kohan, A. F.; Ceder, G.; Cho, K.; Joannopoulos, J. Ab initio study of lithium intercalation in metal oxides and metal dichalcogenides. *Phys. Rev. B* **1997**, *56*, 1354–1365.
